# Supplementary material for: Hybrid Models and Biological Model Reduction with PyDSTool
Source: PLoS Comput Biol. 2012 Aug 9;8(8):e1002628. doi: 10.1371/journal.pcbi.1002628 (PMC3415397; doi:10.1371/journal.pcbi.1002628)
Supplement: Text S4 — Complete source code for the PyDSTool package (version 0.88.120504). Includes API documentation and help files linking to web pages. This file is identical to the current public release on Sourceforge.net. (ZIP) [file pcbi.1002628.s004.zip › PyDSTool/html/PyDSTool.Toolbox.neuro_data.get_spike_model-class.html]

xml version="1.0" encoding="ascii"?


PyDSTool.Toolbox.neuro\_data.get\_spike\_model


| Home | Trees | Indices | Help | PyDSTool |
| --- | --- | --- | --- | --- |

Package PyDSTool ::
Package Toolbox ::
Module neuro\_data ::
Class get\_spike\_model

[hide private]

[frames] | no frames]

# Class get\_spike\_model

source code

```
       object --+            
                |            
 MProject.feature --+        
                    |        
MProject.feature_leaf --+    
                        |    
 MProject.ql_feature_leaf --+
                            |
                           get_spike_model
```

---

Qualitative test for presence of spike in model trajectory data using
events to identify spike times. Also records salient spike information
for quantitative comparisons later.


|  |  |
| --- | --- |
| Instance Methods | [hide private] |

|  |  |
| --- | --- |
| evaluate(self, traj) | source code |

|  |  |
| --- | --- |
| finish(self, traj)  Operations to complete only if evaluate was True (optional). | source code |

**Inherited from `MProject.feature_leaf`**:
`__repr__`,
`__str__`,
`set_ref_traj`

**Inherited from `MProject.feature_leaf`** (private):
`_residual_info`

**Inherited from `MProject.feature`**:
`__call__`,
`__eq__`,
`__hash__`,
`__init__`,
`__ne__`,
`info`,
`postprocess_ref_traj`,
`prepare`,
`reset_metric`,
`validate`

**Inherited from `MProject.feature`** (private):
`_find_idx`

**Inherited from `object`**:
`__delattr__`,
`__getattribute__`,
`__new__`,
`__reduce__`,
`__reduce_ex__`,
`__setattr__`


|  |  |
| --- | --- |
| Properties | [hide private] |

**Inherited from `object`**:
`__class__`


|  |  |
| --- | --- |
| Method Details | [hide private] |

|  |  |
| --- | --- |
| evaluate(self, traj) | source code |

Overrides: MProject.feature.evaluate

|  |  |
| --- | --- |
| finish(self, traj) | source code |

Operations to complete only if evaluate was True (optional). Override
in concrete sub-class if desired

Overrides: MProject.feature.finish: *(inherited documentation)*

  


| Home | Trees | Indices | Help | PyDSTool |
| --- | --- | --- | --- | --- |

|  |  |
| --- | --- |
| Generated by Epydoc 3.0.1 on Fri May 4 15:24:09 2012 | http://epydoc.sourceforge.net |
